# Cellular and Molecular Players in the Interplay between Adipose Tissue and Breast Cancer

**DOI:** 10.3390/ijms22031359

**Published:** 2021-01-29

**Authors:** Francesca Reggiani, Paolo Falvo, Francesco Bertolini

**Affiliations:** 1 Translational Research Unit, Azienda USL- IRCCS di Reggio Emilia, 42123 Reggio Emilia, Italy; Francesca.Reggiani2@ausl.re.it; 2 Laboratory of Hematology-Oncology, European Institute of Oncology IRCCS, 20141 Milan, Italy; paolo.falvo@ieo.it

**Keywords:** obesity, breast cancer, adipose tissue, tumor microenvironment, preclinical models

## Abstract

The incidence and severity of obesity are rising in most of the world. In addition to metabolic disorders, obesity is associated with an increase in the incidence and severity of a variety of types of cancer, including breast cancer (BC). The bidirectional interaction between BC and adipose cells has been deeply investigated, although the molecular and cellular players involved in these mechanisms are far from being fully elucidated. Here, we review the current knowledge on these interactions and describe how preclinical research might be used to clarify the effects of obesity over BC progression and morbidity, with particular attention paid to promising therapeutic interventions.

## 1. Introduction

Obesity, currently defined as an increase in body mass index (BMI), is associated with metabolic disorders, altered production of steroid hormones, chronic tissue inflammation, and an increase in the incidence and severity of several types of cancers [1]. Obesity is a serious problem across the globe: in the United States, nearly two-thirds of adults are overweight or obese [2]. Likewise, in the rest of the world, the percentages are rising dramatically, with projections of nearly 42% of the population being obese by 2030 [3]. Interestingly, most obesity-related cancers arise either within or adjacent to adipose tissue, suggesting a possible crucial role for cellular (in addition to soluble) players [4]. The complexity of these interactions is very intricate, and in some types of cancer and in the case of different therapies, a paradoxical effect has been reported, in which overweight and early obese states are associated with improved survival [5]. In breast cancer (BC), epidemiologic data indicate a positive correlation between overweight/obesity and tumor incidence [6,7,8], together with a worse prognosis [9] and increased resistance to chemotherapy [10].

In this review, we will discuss the role of different cell populations and circulating molecules that might be involved in the interplay between adipose cells and BC, along with preclinical models used for the study of related mechanisms and possible therapeutic interventions.

## 2. Dysfunctional Adipose Cellular Players in BC

**Figure 1:**
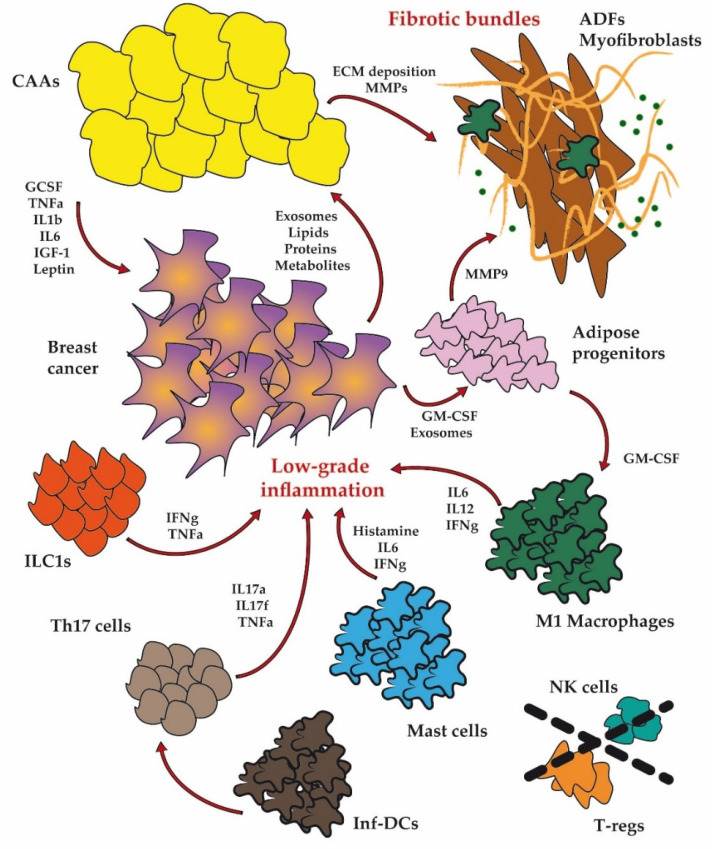
In the case of obesity, white adipose tissue (WAT) is characterized by dysfunctional cellular players, which are interconnected in a network of abnormal regulation and intercellular signaling. Collectively, these alterations and the bidirectional cross-talk with breast cancer cells lead to abnormal extracellular matrix (ECM), fibrosis, low-grade chronic inflammation, and further prompt cancer progression. CAAs: cancer-associated adipocytes; ADFs: adipose-derived fibroblasts; Inf-DCs: inflammatory dendritic cells; ILC1s: innate lymphoid cells type 1; T-regs: T-regulatory cells; NK: natural killer cells.

White adipose tissue (WAT) is a metabolic dynamic organ that is characterized by high cellular heterogeneity and comprises adipocytes, adipose progenitors, fibroblasts, endothelial cells, and infiltrating immune cells. In this section, the principal alterations of WAT cell composition and functionality are summarized in the context of obesity and BC. All of the mentioned dysfunctional cellular players are interconnected in a network of abnormal regulation and intercellular signaling, which are the key determinants of the development of both metabolic disorders and BC (Figure 1).

### 2.1. Adipocytes

The main component of WAT is constituted by adipocytes, which are the reservoirs and sensors of body energy. Adipocytes are functional endocrine secreting cells that are able to release hormones and other paracrine factors into the circulation, thus regulating the whole-body metabolism. In obesity, adipocytes are characterized by a dramatic alteration of the gene expression profile [11,12,13], with relevant implications for tissue and body homeostasis. In obese subjects, WAT is characterized by adipocyte hyperplasia and hypertropia, with a consequent serious metabolic and immune imbalance [14]. Dysfunctional obesity-associated adipocytes share many features with the so-called cancer-associated adipocytes (CAAs) that can be detected at the BC invasive front [15]. CAAs are characterized by de-lipidation, fibroblast-like phenotype, and loss of adipocyte differentiation markers. This dysfunctional phenotype displays an increased secretion of pro-inflammatory cytokines and proteases, such as interleukin (IL)-6 and IL-1B, thus promoting a permissive tumor microenvironment (TME) [15]. The interaction between pre-adipocytes, inflammatory cells, and other stromal cells is crucial in regulating adipocyte expansion and differentiation, deeply influencing the mechanical properties of the expanding tissue, including the presence of fibrotic areas, the down-regulation of brown adipogenesis, and tissue inflammation [16].

A cross-talk exists between BC cells and CAAs, being required in the pro-tumorigenic effect of CAAs compared to naïve adipocytes [15]. BC can modulate adipocyte gene expression by cell-by-cell contact or by the release of proteins, lipids, metabolites, or vesicles (such as exosomes). In turn, CAAs express high levels of granulocyte colony-stimulating factor (G-CSF), which enhances tumor epithelial-to-mesenchymal transition (EMT), migration, and invasion in BC through the activation of Stat3 [17]. Other CAA-released growth factors and cytokines, including tumor necrosis factor (TNF)-α, IL-6, and insulin-like growth factor (IGF)-1, are implicated in the progression of obesity-related cancers [4], as well as adipokines and other lipid metabolites [18,19].

Collectively, the bidirectional cross-talk existing between BC cells and CAAs is one of the main drivers of tumor progression, and this pathological interaction can be even amplified in the case of obesity.

### 2.2. Fibroblasts

Altered tissue remodeling and increased fibrosis are the results of the persistence of the WAT inflammatory process and inadequate angiogenesis [20]. Fibrosis is characterized by excessive extracellular matrix (ECM) deposition by dysfunctional fibroblasts, myofibroblasts, mesenchymal progenitors, and mature adipocytes. Platelet-derived growth factor receptor A (PDGFRα+) progenitors of white adipocytes were recently identified as the main source of ECM deposition [21]. Obese subjects display collagen accumulation around adipocytes, leading to the formation of fibrotic bundles [22,23]. Divoux and colleagues demonstrated that obese subjects have more total and pericellular fibrosis around adipocytes than lean subjects [22]. Indeed, macrophages enrich the fibrotic bundles around adipocytes, further supporting the tight connection with inflammation. Overall, the abnormal fibrotic depot alters the physiological plasticity of WAT, enhancing its stiffness [23]. Consistently, several studies reported a connection between excessive ECM deposition and WAT metabolic and endocrine alterations [24,25,26]. The pathological implication can be expanded to BC onset and progression, since adipose-derived fibroblasts (ADF) are detected near BC cells in clinical specimens, with increased invasive/migratory potential compared to normal fibroblasts [27]. ECM alterations are commonly detected in BC lesions with desmoplasia, further supporting the existence of mechanical niches that are able to promote carcinogenesis and a more aggressive tumor phenotype [24].

The regulation of ECM through matrix metalloproteinases (MMPs) is relevant in both WAT and BC. Several MMPs control adipocyte expansion and differentiation, such as MMP2, MMP3, and MMP14 [28]. MMP9 has been involved in BC invasion and metastatic spread, being a negative prognostic marker and highly expressed in aggressive triple negative breast cancer (TNBC) [29]. Increased production of MMP9 in the TME increases blood vessel formation, ECM remodeling, local tumor invasion, and metastasis in obese mice [30]. MMP-11 is also expressed by WAT when the tumor invades the surrounding tissue, and it is a negative regulator of adipogenesis [31]. The adipocyte dedifferentiation, in turn, promotes the accumulation of peritumoral fibroblast-like cells, which further enhances cancer progression. Overall, these data confirm the existence of a relevant cross-talk between cancer cells and adjacent fibroblast-like cells.

### 2.3. Immune Cells

The immune composition of WAT significantly differs between lean and obese subjects [32]. Lean WAT is enriched with regulatory cells such as T-regulatory cells (T-regs), natural killer (NK) cells, eosinophils, and alternatively activated M2 macrophages. Conversely, the recruitment of pro-inflammatory cells is predominant in obesity, including M1 macrophages and Th-1 cells. Moreover, a time-course experiment in obese mice demonstrated that distinct immune populations act at different stages during obesity progression: T-cell subsets are the early-phase components, whereas pro-inflammatory macrophages and mast cells are the later phase mediators [33].

#### 2.3.1. Macrophages

Macrophages are suspected to be the main players in the adipose inflammatory process, as in obesity, they reach up to 50% of all WAT cells [34]. In lean subjects, macrophages are distributed throughout the tissue with limited inflammatory functions, whereas in obesity, they are located nearby apoptotic/necrotic adipocytes, generating crown-like structures (CLSs) and displaying enhanced proinflammatory features [35,36,37]. The shift of WAT macrophages toward the M1 pro-inflammatory phenotype is driven by interferon (IFN)-γ, which is released by other immune cells, including NK and CD8^+^ T cells. These cells are, in turn, activated by the surface expression of stress markers by dysfunctional adipocytes [38].

A specific “metabolic-altered” phenotype of obesity-associated macrophages has been recently characterized and associated with metabolic dysfunction and nutrient excess [39]. This phenotype is characterized by the absence of classical activation markers, with a paradoxical induction of anti-inflammatory markers, such as peroxisome proliferator-activated receptor (PPAR)-γ [39], and the increased expression of genes involved in lysosome biogenesis and lipid catabolism [40]. Collectively, these data support the hypothesis that macrophages not only contribute to obesity-induced inflammation but have a relevant regulatory role in lipid metabolism.

#### 2.3.2. Dendritic Cells

Resident adipose dendritic cells (DCs) display a distinct role and features in lean individuals compared to obese subjects. Conventional DCs (cDCs) from lean mice retain their presenting cell antigen activity, thus promoting the acquisition of the effector Th1 phenotype by naive CD4^+^ lymphocytes. In obesity, cDCs and atypical DCs, called inflammatory DCs (inf-DCs), accumulate in human and animal WAT [41]. Inf-DCs display a more distinct phenotype than cDCs or plasmacytoid DCs (pDCs). They are characterized by the expression of CX3CR1, which is required to commit naïve CD4^+^ lymphocytes to switch to the Th17 phenotype. The enhancement of Th17 inflammatory response has been associated with the establishment of low-grade chronic inflammation and insulin resistance [41]. pDCs are shown to contribute to the acquisition of obesity-related phenotype, since their depletion in mouse models prevents fat accumulation and insulin resistance [42]. On the contrary, a specific population of DCs, characterized by perforin expression, has been described as a negative regulator of obesity and metabolic syndrome [43]. Therefore, DCs are dynamic players in the context of obesity, and further investigations are needed to clarify the specific contribution to BC of distinct DC subtypes.

#### 2.3.3. Natural Killer Cells

Recent findings suggested that NK cells have a dysfunctional phenotype and impaired functions after being exposed to abnormal levels of adipokines, including leptin, IL-6, and estrogens [44]. The expression of activating NK cell receptors, including NKp46, NKG2D, or NKp30, is impaired in diet-induced obese (DIO) models [45,46], and NK cytotoxicity is markedly reduced in obese models compared to lean animals [47]. Still, the abnormal phenotype may be reverted by transferring NK cells from obese mice to the normal-weight counterparts, suggesting that adipose and the BC environment create a niche that influences NK activities [48]. Obesity has been shown to increase the accumulation of lipids in NK cells due to exacerbation of the PPAR-dependent pathway, causing a drastic impairment of NK trafficking and cytotoxicity [49].

#### 2.3.4. Mast Cells

Another important component of the inflammatory cell compartment is constituted by mast cells, which can release high levels of histamine; proteases; and inflammatory molecules, such as IL-6 and IFN-γ. Mast cells are significantly expanded in serum and WAT from obese subjects, compared to normal-weight counterparts [50]. Activated mast cells are localized nearby fibrosis depots in WAT from obese subjects, with a positive association with macrophage accumulation and inflammation [51]. Accordingly, the depletion or inactivation of mast cells in mouse models prevents body weight gain and ameliorates glucose tolerance [50].

However, conflicting findings reported a different role for mast cells due to the distinct animal model used within each study. Mice carrying Kit-mutant mast cells display important improvement of metabolic parameters [50,52], whereas a different mast cell-deficient strain, which lacks mast cells but expresses a normal level of functional Kit, does not display any effects over metabolic status [52,53,54]. Further studies will be required to clarify the exact role of adipose mast cells in obesity.

#### 2.3.5. T-Regulatory Cells

Adipose T-regs display a distinct molecular pattern compared to other lymphoid organs, mainly characterized by higher PPAR-γ expression [55]. The PPAR-γ level is a key regulator of adipose T-reg number, as shown by the administration of pioglitazone, a known PPAR-γ activator, which significantly enriches adipose T-regs in both lean and obese mice [55]. In several obese mouse models, T-reg frequency and functions are strikingly impaired [56]. Transcriptional profiles of adipose T-regs, isolated from DIO or genetically induced obese mice, differ from lean mice due to the loss of the normal molecular signature associated with adipose T-regs [57]. These cells from obese mice display a novel signature which mainly depends on the functional depletion of PPAR-γ.

#### 2.3.6. Innate Lymphoid Cells

The presence of adipose innate lymphoid cells (ILCs) has been associated with both pro- and anti-inflammatory effects [58]. These cells are currently subdivided into three groups, which are defined by distinct cytokine release and transcription factor expression. The type 1 ILC (ILC1) is characterized by a pro-inflammatory profile, and it has been identified as the prominent WAT resident cell population in case of obesity [58]. 

In contrast, the type 2 ILC (ILC2) has a relevant role in immune–metabolic homeostasis of healthy WAT in lean subjects, contrasting obesity, insulin resistance, and limiting inflammation [59]. The altered balance between ILC1 and ILC2 populations in WAT may be the key driver in obesity, with a progressive conversion of the ILC2 into the ILC1 subtype.

In obesity, only a few studies have investigated the role of the type 3 ILC (ILC3), but it has been hypothesized that IL-17 and IL-22 secreted from ILC3s may favor obesity and inflammation [60]. Still, the total amount of ILC3s is impaired in WAT from obese mice, with the same trend of ILC2s [61].

### 2.4. Adipose Progenitor Cells

The presence of adipose-derived stem/progenitor cells in WAT has been widely reported, as well as their contribution to BC progression [30,62,63]. Adipose progenitors were significantly increased in DIO mice models [62]. This progenitor-like population is composed of two sub-populations with a complementary role in promoting both local and metastatic BC [64]. CD45^−^CD34^+^CD31^+^ Endothelial progenitor cells (EPCs) contribute to the formation of mature endothelial cells and capillaries, support tumor angiogenesis, and enhance cancer cell migration and metastasis. The other population is composed of CD45^−^CD34^+^CD13^+^ adipose stem cells (ASCs) or mesenchymal stem cells, which are able to differentiate into pericytes and to promote local tumor growth. Both EPCs and ASCs induce EMT gene expression in luminal BC cells [63]. The elevated ASC availability in obesity results in the net increase in tumor ASC recruitment, with a 6-fold increase in frequency in obese mice compared to lean mice [64].

The increased availability and activity of adipose progenitors in obesity are likely to trigger the establishment of a permissive TME. ASCs release immune regulatory mediators, such as IL-4, IL-10, and TGFB1, thus promoting immune suppression [65] and T-reg differentiation [66]. Indeed, both ASCs and EPCs release high levels of granulocyte–macrophage colony stimulating factor (GM-CSF) after being exposed to TNBC, supporting their role as immune-regulators [30].

Tumor-induced alterations of adipose progenitors may depend on exosome release, that contain different types of signaling proteins, miRNAs or metabolites. BC-derived exosomes are shown to directly modify ASC phenotype through the acquisition of tumor-associated myofibroblast features [67].

A novel class of adipose progenitors has been recently characterized from their specific molecular signature [68]. This is a subset of CD34-CD29+ cells that is identified as beige adipocyte progenitors. These cells can differentiate into beige adipocytes with unique metabolic and endocrine features, whereas they are depleted in patients with type 2 diabetes. Further studies will be required to clarify their role in obesity and in BC progression.

## 3. Molecular Alterations in Obesity and BC

Obesity-associated WAT is characterized by altered metabolic balance, high oxidative stress, and mitochondrial dysfunction. The physiologic role of adipocytes in energy homeostasis and adipokine secretion is completely lost, as well as the balance of insulin signaling and lipid regulation. The concomitant impaired immune functionality and low-grade chronic inflammation complete the pathological frameshift. The principal molecular alterations occurring in obesity are illustrated in this paragraph to better understand the interconnection with BC initiation and progression.

### 3.1. Inflammation

Inflammation is one of the main drivers of WAT pro-tumorigenic activity, and obesity is associated with a low-grade, chronic inflammation [20]. In BC, both the local and systemic forms of inflammation are negative prognostic markers [69]. Lipolysis and free fatty acids (FFA) release by dysfunctional adipocytes is the primary source of obesity-related inflammation. Obesity leads to the activation of several inflammatory pathways in adipocytes, mediated either by c-Jun N-terminal kinase (JNK) or nuclear factor kappa B (NFκB) [70]. When activated, these pathways increase the release of pro-inflammatory cytokines, which contribute to pro-inflammatory macrophage infiltration. The massive infiltration of macrophages is associated with the release of inflammatory mediators, such as cyclooxygenase-2 (COX-2), TNFα, monocyte chemoattractant protein-1 (MCP-1), IL-1β, and aromatase [1,71], that further trigger the inflammatory loop. NFκB pathway activation in both adipocytes and immune cells plays a critical role in the inflammatory process. Inactive NFκB is sequestered into the cytoplasm, whereas in the presence of various stimuli, NFκB relocates into the nucleus to promote the gene expression of inflammatory mediators, including IL-1, IL-18, TNF-α, and IL-6. Some pro-inflammatory cytokines need to be activated by caspase cleavage, which is dependent on the activation of the inflammasome complex. The inflammasome is an intracellular multimeric complex, which is activated in innate immune myeloid cells in the case of infection [72]. It comprises several nucleotide-binding oligomerization domain (NOD)-containing protein-like receptors (NLRs) that facilitate the activation of pro-inflammatory caspases. The main consequence is the release of a high amount of activated IL-1B and IL-18 by caspase-1. NLRP3 is the principal member of the NLR family and co-localizes with macrophages in CLSs [73]. Genetic ablation of NLRP3 prevents the obesity-induced inflammasome activation in WAT, further clarifying its key role in the process [73].

### 3.2. Metabolism

Obesity is frequently associated with hyperglycemia, insulin resistance, and increased IGF bioavailability [74,75]. The inflammatory mediators produced by dysfunctional WAT spread through systemic circulation, thus activating JNK and NFκB signaling pathways in the liver and skeletal muscle [70]. The normal insulin signaling is dramatically impaired with the down-regulation of the adipocyte insulin-responsive glucose transporter GLUT4 and, in both muscle and adipocytes, the reduction of insulin binding to its receptors [74].

Adipocytes are the main source of autotaxin (ATX), which mediates the production of bioactive lipids called lysophospatides (LPAs). ATX and LPAs have been investigated in several high-fat diet (HFD) mouse models [76,77,78]. Their increase in obesity is mainly associated with adiponectin depletion, a worsening metabolic profile and impaired glucose homeostasis [77]. Both insulin and glucose enhance the ATX release from adipocytes, suggesting that LPA aberrant signaling may be strictly related to obesity-dependent alterations [76]. Adipocyte-derived ATX is also enhanced by the interaction with BC cells, which secrete inflammatory mediators that further prompt the inflammatory loop between cancer cells and adipose tissue [77].

As opposed to WAT, brown adipose tissue (BAT) displays a lower immune cell and macrophage infiltration in DIO mice, suggesting that BAT is more resistant to inflammation [79]. Beige adipogenesis, or “browning”, is a cellular process that regulates insulin sensitivity through the induction of the mitochondrial uncoupling protein-1 (UCP-1) expression in white adipocytes, thus resembling the BAT phenotype [80]. The recruitment of brown adipocytes has been suggested as a therapy to restore the metabolic balance of WAT and ameliorate insulin resistance [81]. Indeed, the transplantation of CRISPR-engineered UCP-1 expressing adipocytes in obese mice restores both the glucose tolerance and the insulin sensitivity, thus reverting the obesity-associated phenotype [82]. Other strategies have been proposed to increase the browning of WAT, such as the deacetylation of PPAR-γ [83] or the ablation of the macrophage IRE1α pathway [84].

Tissue hypoxia is one of the major consequences related to obesity, since oxygen consumption is drastically enhanced during WAT uncontrolled expansion [85]. Hypoxia affects several biological functions, such as adipose angiogenesis, cell proliferation, apoptosis, and inflammation [86]. In obese and diabetic subjects, the mitochondrial oxidative dysfunctions are associated with insulin resistance and correlate with a progressive reduction of the mitochondrial number, size, and enzymatic oxidative capacity [87]. The impaired expression of the oxidative phosphorylation (OXPHOS) genes and the consequent reduced oxygen consumption have also been observed in obese patients [88]. Thus, the accumulation of reactive oxygen species (ROS) by dysfunctional mitochondria leads to a further increase in adipose oxidative stress and a worse hypoxic environment. The hypoxia-induced factor (HIF)-1α is highly expressed in obese WAT, and it worsens inflammation, fibrosis, and insulin resistance in animal models [89]. Overall, obese individuals are more susceptible to oxidative damage compared to lean individuals due to the lack of antioxidant sources and the impaired activity of key enzymes, such as the superoxide dismutase (SOD) [90].

### 3.3. Granulocyte–Macrophage Colony Stimulating Factor (GM-CSF)

Another dysfunctional axis is constituted by the abnormal release of GM-CSF by macrophages and stromal cells, including endothelial cells and fibroblasts. Intriguingly, adipose progenitors can release high levels of GM-CSF in the presence of TNBC and obesity [30]: a positive regulatory loop triggers GM-CSF production upon the presence of tumor cells. Besides its well-known pro-inflammatory role, GM-CSF promotes tumor immune escape through the recruitment of immunosuppressive cells, namely T-regs, myeloid-derived suppressor cells (MDSCs), tumor-associated macrophages (TAMs) [30], and neutrophils [91]. GM-CSF has been identified as a crucial factor in the establishment of the pre-metastatic niche in lungs during BC progression [91]. In this model, the plasmatic levels of GM-CSF are significantly increased in mice with higher adiposity and directly correlated with tumor growth. Indeed, the generation of a pre-metastatic niche in WAT-poor organs might be a direct consequence of the imbalanced secretion of GM-CSF in the adipose tissue surrounding the primary neoplastic lesion [92].

Recently, the pleiotropic function of GM-CSF has been further extended, as its depletion from DIO mice prevents the development of insulin resistance [93].

### 3.4. MiRNAs

A relatively novel approach to the field identifies the small non-coding RNAs or microRNAs (miRNAs) as key regulators of obesity and cancer [94]. The principal role of miRNAs in WAT is the control of adipocyte differentiation and functions, including metabolic and endocrine activities [95]. Several plasmatic miRNAs are detected tremendously up-regulated in the circulation of obese subjects compared to lean individuals [96,97]. Accordingly, many miRNAs are detected significantly up- or down-regulated in WAT or liver of DIO or genetic-induced obese animal models [94]. 

Among the obesity-related up-regulated miRNAs, several are involved in insulin and glucose homeostasis: miR-221/222 are shown to negatively regulate adiponectin receptor 1 (ADIPOR1), which promotes insulin sensitivity [98], and induces EMT in BC [99]; miR-520e and miR-141 specifically control glucose and lipid homeostasis [100]; miR-4454 regulates splicing variants of insulin receptors [97]. Conversely, other miRNAs are down-regulated in obesity, such as miRNA-592, which targets the FOXO1 transcription factor and ameliorates hyperglycemia [101]. Dysregulated miRNAs have also been correlated with the control of WAT inflammatory response through the regulation of both adaptive and innate immunity, TNFα, and NFκB pathways [102,103].

The clinical relevance of obesity-associated miRNAs has been recently speculated in BC [104,105]. MiRNAs represent attractive therapeutic targets that can be exploited either to specifically hit dysfunctional WAT, as well as useful diagnostic/prognostic biomarkers for cancer patients through an obesity-associated “miRNA signature” [106].

## 4. Preclinical Models to Study Obesity

Given the serious consequences of obesity to human health, model organisms that faithfully recapitulate obesity and its effects are urgently needed. Most models were created in rodents [107]. Rodents used for obesity and metabolic experiments are commonly divided into two main categories: (a) Genetically modified rodents: animals bearing mutations in one of the key genes that regulate food uptake or the appetite/satiety cycle (e.g., leptin mutated mice) [108]; (b) DIO rodents: animals fed with a diet containing augmented amounts of fatty acids and cholesterol [109].

The decision regarding the use of genetically modified or DIO models depends on the experimental goal. The first category is mainly used to dissect metabolic pathways and to study possible therapeutic approaches. Instead, DIO models are used to investigate mechanisms correlating obesity with a given pathology, such as cardiovascular disease or cancer. 

This paragraph aims to provide an in-depth description of genetically modified and DIO animals.

### 4.1. Genetically Modified Obese Models

Mutations in several genes play a role in the onset of spontaneous obesity. Proteins encoded by implicated genes control pathways involved in metabolism or perception of satiety. It should be pointed out that in these models, even a single mutated gene is enough to induce obesity, whereas in humans, the cause is frequently polygenic [107]. Thus, the genetic make-up of model organisms does not reflect completely that of humans. 

One of the most studied pathways in the history of obesity research is the leptin cascade. Mutations in the leptin gene, named *ob*, were first observed in 1950 in mice [110]. Scientists found that these mice had an increased appetite and became obese spontaneously. It took 40 years for the molecular mechanism to be elucidated [111]. However, mutations in the orthologue gene rarely lead to obesity in humans [112]. Leptin is a small protein released by WAT and acts in the brain. Leptin levels fluctuate in the blood according to changes in calorie intake: they normally decrease during starvation and an increase upon food intake [113]. Once released by WAT, leptin binds its receptor (named LepRb) in the hypothalamus and, herein, initiates a cascade by activating transcription factors such as STAT3 [114]. In particular, leptin receptors are enriched in two different regions of the hypothalamus: the arcuate nucleus (ARC) and ventromedial hypothalamus (VMH) [114]. The ARC is composed of two neuron populations: the first co-expresses agouti-related peptide (AgRP) and neuropeptide Y (NPY), whilst the other expresses proopiomelanocortin (POMC) and cocaine- and amphetamine-regulated transcript (CART). The two populations have opposite functions: the first stimulates the sense of appetite, favoring food intake, while the latter stimulates the perception of satiety, resulting in reduced food intake. These neurons are key targets for leptin, which inhibits NPY/AgRP and stimulates POMC/CART neurons, leading to a reduction of food intake, increased energy expenditure, and a decreased body weight [114]. 

There are murine strains with spontaneous mutations in the leptin gene, or its receptor. Mice are named Lep^ob^/Lep^ob^ and Lep^db^/Lep^db^, respectively. They both show hyperphagia and insulin resistance and, consequently, develop obesity. Compared to Lep^ob^/Lep^ob^, Lep^db^/Lep^db^ are completely leptin resistant due to the mutation in the receptor, despite the fact that their blood contains high levels of leptin. As for the *ob* gene mutation, leptin receptor gene mutations are poorly associated with obesity in humans. Other than spontaneous mutations, mice were genetically engineered to develop obesity by disrupting STAT3 function. The protein is activated after leptin binding to its receptor and mediates the effect of leptin in the brain [108]. STAT3 mutated mice, named s/s mice, develop obesity as efficiently as spontaneous mutated models. Compared to Lep^ob^/Lep^ob^, genetically modified s/s mice are fertile and are less hyperglycemic than the Lep^db^ /Lep^db^ mice [115,116]. Taken together, all three models described above are suitable to study insulin resistance and hyperglycemia. 

Similarly, in rats, only mutations in the leptin receptor were identified. Rats with mutations are called obese Zucker and Koletsky rats and develop hyperphagia and insulin resistance [117,118]. The main difference between the two types regards the molecular mechanism, by which leptin receptor expression is inhibited. In the case of obese Zucker rats, the mutation prevents the expression of the protein on the cell surface, while in the Koletsky rat, there is a stop mutation that disrupts protein structure and functionality [117,118].

Similarly, downstream mutations in the leptin cascade can lead to obesity. Leptin activates the POMC/CART and represses NPY/AgRP neurons, stimulating the perception of satiety. Mutations in this axis can also lead to the spontaneous development of obesity. POMC is the precursor of different molecules, such as α-melanocyte-stimulating hormone (αMSH). This molecule reduces the appetite and stimulates energy consumption by activating melanocortin (MC) 3 and 4 receptors in the hypothalamus. Lack of POMC protein in mice leads to the spontaneous development of obesity with insensitivity towards leptin [119]. However, the phenotype can be restored by αMSH or with an agonist of MC3 and MC4 receptors, such as MC II administration [120]. Along with POMC inhibition, the knockdown of MC4 and MC3 proteins in mice gives rise to the obese phenotype. Specific inactivation of the MC4 receptor produces hyperphagia and morbid obesity [121]. Mice do not respond to leptin or αMSH. Differently, MC4 mutations are thought to be responsible for the obese phenotype in humans. Mice lacking the MC3 receptor, instead, are obese with a late onset of the pathology [122]. Finally, researchers genetically modified mice co-expressing truncated forms of MC3 and MC4 proteins (MC3^−/−^ and MC4^−/−^ double knock-out mice); such mice have an obese phenotype, and they are insensitive to leptin and αMSH but sensitive to the MC agonist MT II [123].

### 4.2. Diet-Induced Obese (DIO) Models

DIO mouse models have attracted much attention from the scientific community in recent years. Model animals are fed with diets rich in fatty acids and cholesterol, or even with diets in which particular components are enriched (e.g., increased amount of any given fatty acid). The main advantage of this approach is the development of obesity over a longer time, which allows for the analysis of obesity at the initial, intermediate, and final phases. In this temporal window, it is also easier to study the correlation of obesity with the onset of other pathologies, such as cardiovascular diseases and cancer [107,124]. Compared to genetic models, DIO models mimic in a more realistic manner what happens in humans. Since in humans, the genetic component of obesity is polygenic and impacted by both the environment and the diet, a mouse model in which fat accumulation derives from an unbalanced diet is more physiological. Moreover, the diet can be changed from a high-calorie to a low-calorie diet to study the effects of slimming on the phenotype. This has a strong potential to monitor whether diet changes can prevent or attenuate the disease phenotype. However, there is a caveat to DIO models in mice. Not all mice strains have the same sensitivity towards the diet; some of them are actually called diet-resistant (DR) mice [125]. For example, the BALB/c strain, for reasons not completely understood, is quite insensitive towards HFD. This obviously limits the possibility to reproduce all the experiments in all strains [126]. 

Several diets have been proposed by researchers. A great example is offered by the so-called “cafeteria diet”. This approach was firstly tested in the 80s by Rothwell and colleagues [127,128,129]. The name refers to the high-sugar, high-calorie, and highly palatable food that are normally served in cafeterias. They firstly created a list of 40 foods, and every day, for a total of 15 days, they provided animals with 4/40 food items. They observed hyperphagia in rats. However, animals compensated for the body weight increase by activating BAT thermogenesis. Although the effect was straightforward, the experiment was highly criticized because of its low-reproducibility level. The use of a diet that is so heterogeneous limits its reproducibility [130]. 

Another interesting approach is the binge-type feeding. Binge-eating behavior is typically observed in people suffering from eating disorders (such as anorexia or bulimia) or in obese people. It consists of food consumption in a shorter time with a loss of sense of control [131]. Binge episodes can occur quite frequently (weekly or monthly) and are triggered by stress or psychological disturbance. People are so stressed that they vent their anger by eating compulsively [132]. This problem has strong consequences in terms of health because binge-eating people gain weight and display a notable increase in BMI. Moreover, it was observed that binge eating has strong consequences on the psychological stability of people [133,134]. It was estimated that adolescents suffering from binge eating have more suicidal thoughts and attempts than healthy adolescents [135]. Since this is a concrete problem with series of repercussions on health, researchers tried to mimic this behavior in mice. Normally, food is given to mice ad libitum, meaning that animals can eat 24 h a day. Researchers developed the binge-type feeding approach, which consists of feeding animals with particularly high-fat food in a limited time and normally when animals are not hungry. For example, they provided a high-fat diet only for two hours, and for the rest of the time, they provided a standard chow diet [136,137]. Strikingly, they observed that half of the calories were taken during the HFD period. However, rodents were shown to respond differently to binge eating: rats absorbed 50% of their calories during a binge diet period with a consequent increase in body weight, while mice absorbed more calories (almost 86%) in the same time, but with a lack of increase in body weight [138,139].

## 5. Obesity and BC in Preclinical Models and Therapeutic Approaches

Mounting evidence from the 1950s correlates obesity with the risk of developing spontaneous BC in preclinical models [140,141]. Subsequent experimental studies further support the close link between obesity and postmenopausal BC in mice, especially in the case of estrogen receptor positive (ER^+^) and progesterone receptor positive (PR^+^) tumors [142,143]. 

Still, understanding how obesity influences BC onset is an open question. Obesity may accelerate the onset of cancer through different mechanisms, which may be context- and tumor-dependent, dynamic, and difficult to reproduce in vitro. Therefore, only complex model systems might unravel how obesity influences cancer onset. Genetically engineered mouse models that recapitulate physiological changes and characteristics of obese subjects are well established, being largely used to investigate the association with BC [144,145].

In the next paragraph, recent breakthroughs in this field will be examined with particular attention to the therapeutic approaches.

### 5.1. Investigation of Obesity in Murine Models of BC

Preclinical models of hormone receptor positive BC are generally more used due to the stronger association observed between obesity and BC risk in post-menopausal women [146] (Table 1). The relative contribution of obesity to post-menopausal BC has been investigated in ovariectomized C57BL/6 J mice, which were fed either with a regular diet or HFD [147]. BC from obese mice displays higher grade and EMT. Of interest, HFD tumors were transplanted into RD mice but maintained the same pro-metastatic phenotype with an enhanced BC growth and lung metastasis formation [147]. These findings support the notion that the more aggressive behavior of BC cells is primed by obesity and its TME. The hypothesis that obesity deeply influences TME in which the tumor resides is corroborated by our works in preclinical models [30,62,63] and deserves to be further investigated.

The direct comparison of the role of obesity in pre- or post-menopausal BC has been analyzed by Cranford and colleagues [148]. PyMT/MMTV ovary-intact or ovariectomized mice, which were fed with regular or HFD diet, were compared for BC progression and molecular characteristics. HFD pre-menopausal (ovary intact) mice displayed increased adiposity and increased BC progression, assessed by histopathological scores. Moreover, a significant association with WAT aromatase and macrophage marker expression was detected. Conversely, HFD had no similar significant effect on tumorigenesis in postmenopausal mice (ovariectomized), even when larger adiposity was observed. However, in a different model of postmenopausal BC (MMTV-TGF-α), the HFD increased adiposity and shortened BC latency [149,150]. High aromatase and estrogen levels are associated with an increased risk of BC in obese postmenopausal models [151]. Accordingly, the DIO syngeneic mouse model of BC (C57BL/6 strain) showed an increased tumor rate, specifically in ovariectomized mice [143].

Genetically modified obese mice have also been used to investigate the association between obesity and BC, with particular attention paid to leptin involvement and its controversial activity [152,153]. Leptin-deficient obese mice (Lep^ob/ob^) did not develop spontaneous BC, suggesting that they are not the ideal model [152]. In contrast, Lep^db/db^ mice displayed a significant outgrowth of BC upon syngenic transplantation, whereas Lep^ob/ob^ mice exhibited an impaired tumor growth due to the drastic reduction of tumor-initiating cells [153].

Overall, these findings suggest a complex role of obesity in BC initiation and progression that cannot be generalized due to the intrinsic limitations within each mouse model (Table 1).

### 5.2. Therapeutic Interventions

Preclinical studies to treat obesity may be performed targeting metabolic enzymes or/and the perception of hunger and satiety. This offers a new layer of difficulty in the treatment of pathological obesity, since it involves not a specific gene but neural circuits. Therefore, the majority of drugs that have been recently proposed to treat obesity have been withdrawn due to their inefficacy [107].

Caloric restriction (CR) is a common method that induces the reversion of obesity-dependent pro-tumorigenic effects, including systemic and mammary gland inflammation in murine models [161,162]. The combination of CR with exercise has been shown to ameliorate the deleterious effects of obesity in mouse models, regulating distinct pathways and genes that are involved in the control of BC stem cells in the mammary gland, EMT, and BC proliferation [163]. However, weight normalization could not be enough to completely reverse obesity effects due to the persistence of epigenetic reprogramming and inflammatory signals in TME [154].

Another approach that has been shown to be effective in mitigating the adverse effects in the case of ER^+^ tumors is the administration of vitamin D, which inhibits aberrant estrogen synthesis and signaling in obese mice [155].

Despite the disappointing results obtained with anti-angiogenic therapies as anti-neoplastic treatments [164], obese patients may benefit from anti-angiogenetic drugs in BC progression, although obesity has been shown to increase anti-VEGF therapy resistance [156]. The inhibition of adipocyte-derived angiopoietin-like 4 through neutralizing antibodies significantly impaired tumor angiogenesis and progression in obese models [157]. Metformin is a biguanide drug with hypoglycemic activity, and its administration to mouse models has a potent anti-angiogenic and anti-tumor activity in vivo [30,165,166]. 

Another therapeutic intervention is the modulation of obesity-related systemic and WAT inflammation. Metformin has been shown to inhibit aromatase expression and macrophage accumulation in obese rats, being able to reduce post-menopausal BC progression [167]. The administration of omega-3 polyunsaturated fatty acids significantly reduced inflammation, insulin resistance, and BC growth in obese models [158]. Resveratrol, a vegetal anti-inflammatory compound, prevented obesity-associated inflammation, reducing BC proliferation, adipocyte hypertrophy, macrophage infiltration, CLS prevalence, and serum cytokines [159].

The poorer therapy outcome that characterizes obese patients may be due to the limited chemotherapy [168] they received when compared to normal-weight subjects. Obesity may also affect chemotherapeutic pharmacokinetics through the increased drug distribution volume due to the excess of WAT [169]. The increased occurrence of innate or acquired drug resistance mechanisms is another issue. CAAs protect cancer cells from doxorubicin by the release of resistin which dramatically impairs tumor apoptosis through the induction of autophagy [170]. The up-regulation of membrane drug-transporter proteins on tumor cells is another common drug resistance mechanism. CAAs enhance BC expression of transport-associated major vault protein (MVP), thus impairing doxorubicin efficacy, which is more amplified in obese patients [171]. Response to trastuzumab in Her2^+^ BC was strongly inhibited by the abundance of adipocytes and pre-adipocytes, which inhibited NK cell cytotoxicity and reactivity against tumor cells in mouse models [172]. 

In contrast, Wang and colleagues demonstrated that checkpoint inhibition success is higher in obese mice and obese cancer patients, suggesting that the checkpoint blockade directly targets some of the immune-related pathways that are dysfunctional in obesity [160].

Taken together, these studies point out a key role of obesity in define the success or failure of anti-neoplastic treatments. Further studies are still required to refine targets, murine models, and strategies to improve therapy outcome of obese patients.

## 6. Conclusions

Obesity-related metabolic and inflammatory changes in WAT are known to disrupt physiological homeostasis, both within local tissues and systemically. These changes promote the incidence and severity of BC, meaning that obesity is currently estimated to contribute to up to 20% of cancer-related deaths [9]. WAT cells and obesity-induced circulating molecules are at the center of a very complex net of interactions involving, among others, inflammation, angiogenesis, and fibrosis. The targets of future prevention and therapeutic interventions in oncology should include strategies to normalize these derailed interactions and to develop proper preclinical models.

## Figures and Tables

**Table 1:** Overview of the main features of preclinical mouse models employed to investigate the interplay between obesity and breast cancer (BC).

Mouse Model	Immuno-Competent	BC Subtype	Obesity Model	Menopausal State	Reference
FVB/N	Yes	MMTV-ErbB2+ (Her2+)	DIO	pre	[30]
BALB/c	Yes	4T1 (ER−, PR−, Her2−)	DIO	pre	[30]
BALB/c	Yes	4T1 (ER−, PR−, Her2−)	DIO	post	[46]
BALB/c Nude	No	MCF-7 (ER+)	DIO	pre	[47]
RAG-1	No	MDA-MB-231 (ER−, PR−, Her2−)	DIO	pre	[64]
C57BL/6	Yes	MMTV-PyMT (99LN, 86R2 Her2+)	DIO	pre	[91]
C3H	Yes	Spontaneous	Chemical induced (thioglucose)	post	[140,141]
C57BL/6	Yes	MMTV-Wnt1 (basal-like, ER−, PR−, Her2−)	DIO	post	[143]
FVB/N	Yes	MMTV-rtTA;TetO-HER2/neu (Her2+)	DIO	pre	[144]
C57BL/6	Yes	E0771 (luminal B, ER+, Her2+, PR+); Py230 (luminal, ER+/−, PR+/−)	DIO	post	[147]
FVB/N	Yes	MMTV-PyMT (Her2+)	DIO	post	[147]
C57BL/6	Yes	MMTV-PyMT (Her2+)	DIO	pre and post	[148]
C57BL/6	Yes	MMTV-TGF-α (ER+)	DIO	post	[149,150]
C57BL/6	Yes	MMTV-TGF-α (ER+)	Genetic (Lep^ob/ob^)	pre	[152]
C57BL/6J	Yes	MMTV-Wnt1 (basal-like, ER−, PR−, Her2−)	Genetic (Lep^ob/ob^; Lep^db/db^)	pre	[153]
C57BL/6	Yes	MMTV-Wnt1 (basal-like, ER−, PR−, Her2−)	DIO	pre	[154]
C57BL/6	Yes	MMTV-Wnt1 (basal-like, ER−, PR−, Her2−)	DIO	post	[155]
C3H; C57BL/6	Yes	E0771 (luminal B, ER+, Her2+, PR+)	DIO	pre	[156]
C57BL/6N	Yes	Py8119 (ER−, PR−, Her2−); E0771 (luminal B, ER+, Her2+, PR+)	DIO	pre	[157]
C57BL/6J	Yes	Py230 (luminal, ER+/−, PR+/−)	DIO and genetic (Lep^ob/ob^)	post	[158]
C57BL/6	Yes	MMTV-Wnt1 (basal-like, ER-, PR−, Her2−)	DIO	post	[159]
BALB/c	Yes	4T1 (ER−, PR−, Her2−)	DIO	pre	[160]

DIO: diet-induced obesity.
